# Incorporation of a Case-based Health Equity Curriculum into Morbidity and Mortality Conference

**DOI:** 10.5811/westjem.2022.11.57723

**Published:** 2022-12-21

**Authors:** Jossie A. Carreras Tartak, Giovanni Rodriguez, Eric Goralnick, Wendy L. Macias-Konstantopoulos, Daniel J. Egan

**Affiliations:** * Massachusetts General Hospital, Department of Emergency Medicine, Boston, Massachusetts; † Brigham and Women’s Hospital, Department of Emergency Medicine, Boston, Massachusetts

## BACKGROUND

The emergency department (ED) serves as a safety net for vulnerable populations that might otherwise be unable to engage with the healthcare system, including patients who are underinsured or who might feel discriminated against based on their social identity.^1^–^2^ To provide the highest quality of care emergency physicians should have the structural competency to identify and address the potential barriers to care that their patients may face. The Accreditation Council for Graduate Medical Education (ACGME) requires that residents “receive training and experience in quality improvement processes, including an understanding of health care disparities.”^3^

Advances within emergency medicine (EM) education have included dedicated curricula on social determinants of health, implicit bias, structural racism. and issues affecting the LGBTQ+ community.^4^–^8^ Many of these initiatives have been targeted toward trainees, most of whom have had some formal training on these topics during medical school compared to previous generations that may not have received similar training.^9^ Although these advances exist, it is unclear how many programs use the curricula, and there are no longitudinal health disparities curricula for EM. Therefore, education on health disparities can be variable across institutions, and past surveys of residents across different specialties have reported low preparedness to deliver cross-cultural care.^10^,^11^

Furthermore, many of the educational advances consist of brief interventions rather than longitudinal initiatives.^5^,^6^ For example, residents within our program led a three-hour health equity retreat during didactic conference that focused on racism and microaggressions.^7^ Building on the success of this health equity retreat in our program, we sought to expand on it and develop a longitudinal and sustainable curriculum.

Integration of health disparities’ education into morbidity and mortality (M&M) conferences was successfully piloted at the University of Michigan and University of Maryland general surgery residencies, and our aim was to trial a similar structured curriculum during our M&M conference while adding unique survey data to examine the ability to respond to discrimination.^12^

The M&M conference is a highly attended portion of our residency’s weekly didactic conference, providing a wide audience that includes faculty members who might otherwise not receive formal training on healthcare disparities as well as advanced practice providers (APP) and nurses. Furthermore, M&M provides an established framework to analyze cases from a quality and safety perspective, shifting the focus from individual physicians to system factors contributing to errors or adverse patient outcomes. Human factors related to communication, attitudes, and environmental culture also impact care.^13^,^14^ Approaching cases through a systems-based and human factors approach can help introduce potentially sensitive topics, such as racism and implicit bias, and shift the focus of the discussion to the structural inequalities that lead to health disparities rather than interpersonal or cultural factors.^15^

## OBJECTIVES

The educational objectives of our curriculum for EM residents and faculty were the following: 1) to understand the disparities that affect our patient population; 2) to understand the disparities that affect colleagues who are underrepresented in medicine; 3) to identify instances of discrimination in the workplace as they affect both patients and clinicians; and 4) to apply the lessons learned through our curriculum when addressing these instances of discrimination.

## CURRICULAR DESIGN

Our Health Equity Curriculum (HEC) was developed using Kern’s curricular design framework.^16^ A consensus group of six residents from our Social EM Interest Academy and five senior faculty members (program director; assistant program director; vice chair of diversity, equity and inclusion; and others with research and academic interests related to equity) met over four months (May-September 2020) to identify curriculum gaps using the majority decision rule. Topics were identified based on a literature review as well as an informal needs assessment through which all faculty and residents were invited to contribute topics via email that they would like to see covered in the curriculum. A health equity email account was developed to solicit cases from residents and faculty members and moderated by HEC leadership. This email account was used both to forward cases for formal quality review by departmental leadership mirroring all other safety reporting and to identify potential future topics. Cases of interest demonstrated how structural inequalities and biases were involved in potential or realized adverse outcomes, such as delays in care or missed diagnoses. Topics were identified for monthly delivery over a 24-month cycle (Table 1), so that they would be repeated twice in the four-year training period of each resident.

The HEC leadership met with M&M and departmental leadership to strategize the delivery format of the case-based sessions, opting to extend M&M conference from 60 to 90 minutes to accommodate a 30-minute health equity topic either before or between M&M cases. The HEC leadership created a standard lecture template to mirror the format of M&M conference. Each lecture began with a de-identified case of a patient or physician experiencing an inequity that aligned with the session’s topic. Cases were primarily sourced from our HEC email account. Sessions concluded with a discussion of systemic factors that contributed to that incident, a literature review on the topic, and action items to prevent or address these incidents when they happen. Interested residents prepared sessions with the mentorship of self- or peer-identified faculty members with subject expertise, as defined by either prior technical, teaching, or research experience in the relevant topic. Monthly HEC case reviews and lectures were collected via anonymous surveys sent to all faculty members and residents in our program after six months of the curriculum being in effect. The Mass General Brigham Institutional Review Board deemed this survey to be exempt from review.

## IMPACT/EFFECTIVENESS

With 18 sessions completed, we have demonstrated the feasibility of incorporating health equity education into M&M conference within our residency program. This approach has led to the incorporation of race and ethnicity, language, insurance status, and contribution of structural inequities in all M&M cases. It has also led to an established workflow for identifying and addressing cases where structural inequalities led to adverse outcomes within our department.

For our six-month survey, we had a total of 38 respondents (14/60 residents and 24 faculty members). Most respondents (97%) reported that the length of the lectures was “just right.” Respondents were split regarding the timing of the lectures, with 16 (42.1%) expressing a preference for the HEC lectures to take place before M&M cases, 16 (42.1%) between cases, and five (13.1%) after cases. Using a five-point Likert scale, respondents were asked to rate their level of agreement with a series of statements (Table 2). Respondents on average agreed that the HEC lectures increased their understanding of the disparities that affect our patients and our colleagues and increased their ability to identify instances of discrimination in the workplace and respond to them.

Several respondents commented that these sessions empowered them on shift to address instances of discrimination with data to support their actions and a plan on how to act. Respondents identified areas of opportunity, such as expanding the curriculum to involve input and participation from other health professionals, (eg, nurses and APPs) and developing a repository of health equity resources that can be easily accessed on shift.

Limitations in assessment of the curriculum included the inability to calculate an accurate survey response rate given that the survey was sent to all faculty members and residents rather than to those who specifically attended at least one or more HEC sessions. We do know that given our total number of residents and faculty at two sites that the response rates were low. Additionally, survey results were self-reported rather than evaluative of attainment of the curriculum learning objectives. This is further limited by the lack of pre- and post-testing, as most residents and younger faculty have had other education related to health disparities outside our curriculum.” Self-reported survey results may also have been influenced by social desirability bias, as many of these issues had more public discourse and respondents may have been primed to more positively review these interventions and topics. Lastly, members of the HEC consensus group were not explicitly excluded from the feedback survey, and their responses could have overstated the impact of this curriculum on our program.

Another factor that could limit the generalizability of this curriculum is the wide variation of M&M conference across institutions. Our program has the benefit of two sites with close to 120 faculty members, providing many potential mentors. However, even without faculty experts, the content is evidence-based and generalizable and could be shared with minimal new development by others. To succeed the curriculum will also require support of residency, departmental, and quality leadership to modify M&M conference.

## CONCLUSION

This Health Equity Curriculum delivered through case-based discussions in morbidity and mortality conference contributes to the limited existing literature regarding education in structural competency, while also providing a novel and sustainable approach to integrating formal education on health disparities into resident education and faculty continuous medical education. The case-based nature of the HEC makes it possible for other programs to replicate the curriculum while individualizing it to meet local health equity needs and concerns. Further research is needed to determine whether such a curriculum can lead to improved knowledge and changes in practice that can mitigate disparities facing our patients and colleagues who are underrepresented in medicine.

## Figures and Tables

**Table 1:** List of covered Health Equity Curriculum (HEC) topics.

Month	Topic	Educational goals
1	Introduction to curriculum	To provide a 10-minute overview of the HEC and its educational goals at faculty meeting at both academic medical centers and during resident conference.
2	What is race and the implications of structural racism in medicine	To understand race as a social construct and the foundational ideas of critical race theory and its importance in medicine. To identify examples of structural violence and to understand the impact of structures on healthcare. To recognize the process of naturalized inequality and the implicit frameworks which justify the perpetuation of structural racism by healthcare providers.
3	History of racism in medicine	To understand the role that medicine played in constructing racial categories, the historical legacy of medical and scientific experimentation on Blacks, and how such a legacy impacts bias and trust in medicine today.
4	Biases against patients with substance use in the ED	To discuss the physiologic nature of substance addiction, genetic factors, and social circumstances that predispose patients to substance use, and behavioral interventions that may be effective at helping these patients.
5	Intersectionality and its role in patient care	To recognize the impact of compounding biases and overlapping systems of oppression against “minority” groups (eg, non-White races, sexual and gender diversity, women) and, thus, the complex intersection of anti-racism, LGBTQ+ affirmation, and feminist frameworks.
6	Racial disparities in ED restraint use	To discuss disparities in care that patients with mental health conditions face. To discuss the intersectionality of mental health disparities (eg, patients of color and non-English speaking patients are more likely to be restrained). To discuss de-escalation measures to avoid restraints.
7	Healthcare disparities in immigrant populations	To discuss the unique challenges of the immigrant population, including language barriers, limited social resources, difficulty navigating social systems, and unique working conditions predisposing them to abuse and occupational hazards.
8	Racial disparities in ED diagnosis and treatment	To discuss actionable racial disparities affecting patients in the ED, reviewing literature on disparities in stroke care and delays in IV access.
9	Gender schemas and their role in the ED	To define gender schemas and how they affect the professional development of women in medicine as well as the care received by female patients.
10	LGBTQ+ health disparities	To introduce healthcare disparities of the LGBTQ+ population and the impact on ED care.
11	Treating gender diverse and transgender patients in the ED	To introduce proper terminology and discuss definitions of gender identity, sexual orientation, and romantic preferences. To highlight specific disparities in care of the transgender population.
12	Racial Disparities in ED Triage	To understand the racial and ethnic biases in emergency triage, how they affect patients, and ways to address those biases.
13	Considerations in the Care of Undocumented Patients	To understand the barriers to healthcare that undocumented patients face in the United States. To equip clinicians with tools to help undocumented patients navigate the health system.
14	Racial disparities in the care of pregnant patients	To understand the racial disparities in outcomes of pregnant patients and ways in which they can be connected to resources in an emergency setting.
15	Biases in the care of mental health patients	To discuss how biases against mental health patients can lead to missed diagnoses and adverse outcomes.
16	Caring for patients with disabilities	To equip clinicians on accommodating the needs of patients with different disabilities in an emergency setting.
17	Capacity assessments and disparities in care of patients with dementia	To equip clinicians with the tools to properly evaluate capacity in patients with altered mental status or cognitive decline. To discuss disparities in the care of patients with dementia as well as the intersection of race and ethnicity in contributing to those disparities.
18	Religious considerations in emergency care	To discuss religious considerations that can affect the care patients receive in the emergency setting.
19	Caring for incarcerated patients	To discuss how biases against incarcerated patients or patients otherwise involved with the legal system can lead to adverse outcomes.
20	The role of ageism in the emergency department	To introduce ageism and explore its manifestations in our department. To discuss ways to improve care for our geriatric population.
21	Socioeconomic disparities in emergency care	To discuss bias towards patients experiencing homelessness or other forms of financial hardship. To discuss biases in perceptions of patients’ appropriate use of resources.
22	Trauma-informed care in the emergency department	To understand the impact of trauma, recognize signs and symptoms of trauma, appropriately respond to patients with trauma, and avoid re-traumatization.
23	Diversity in medicine: the evidence and benefits	To define diversity and discuss the evidence-based, practical benefits of diversity, particularly in patient outcomes.
24	Disparities in the treatment and evaluation of URM trainees	To discuss disparities in how URM residents and attendings are evaluated by supervisors, colleagues, and patients.

*ED*, emergency department; *LGBTQ*, lesbian, gay, bisexual, transgender, queer or questioning; *URM*, underrepresented in medicine.

**Table 2:** Average Satisfaction Scores on 6-month survey

On a scale of 1 to 5 with 5 being “strongly agree”, please rate your level of agreement with the following statements	Faculty members (n = 24)	Residents (n = 14)
The HAEMR Health Equity Curriculum has increased my understanding of the healthcare disparities that affect my patients.	4.04	4.21
The HAEMR Health Equity Curriculum has increased my understanding of the disparities that affect my colleagues who are underrepresented in medicine.	4.13	4.21
The HAEMR Health Equity Curriculum has increased my ability to identify instances of discrimination in the workplace.	3.88	4.29
The HAEMR Health Equity Curriculum has increased my ability to respond to instances of discrimination in the workplace.	3.96	4.21

*HAEMR*, Harvard Affiliated Emergency Medicine Residency
